# RNA-guided transcriptional activation via CRISPR/dCas9 mimics overexpression phenotypes in Arabidopsis

**DOI:** 10.1371/journal.pone.0179410

**Published:** 2017-06-16

**Authors:** Jong-Jin Park, Emma Dempewolf, Wenzheng Zhang, Zeng-Yu Wang

**Affiliations:** 1 Forage Improvement Division, The Samuel Roberts Noble Foundation, Ardmore, Oklahoma, United States of America; 2 BioEnergy Science Center, Oak Ridge National Laboratory, Oak Ridge, Tennessee, United States of America; National Institutes of Health, UNITED STATES

## Abstract

Clustered regularly interspaced short palindromic repeats (CRISPR) and the CRISPR associated protein 9 (Cas9) system allows effective gene modification through RNA-guided DNA targeting. The Cas9 has undergone a series of functional alterations from the original active endonuclease to partially or completely deactivated Cas9. The catalytically deactivated Cas9 (dCas9) offers a platform to regulate transcriptional expression with the addition of activator or repressor domains. We redesigned a CRISPR/Cas9 activation system by adding the p65 transactivating subunit of NF-kappa B and a heat-shock factor 1 (HSF) activation domain to dCas9 bound with the VP64 (tetramer of VP16) activation domain for application in plants. The redesigned CRISPR/Cas9 activation system was tested in Arabidopsis to increase endogenous transcriptional levels of *p**roduction of* *a**nthocyanin* *p**igment 1* (*PAP1*) and *A**rabidopsis thaliana* *v**acuolar H*^*+*^-*p**yrophosphatase* (*AVP1*). The expression of *PAP1* was increased two- to three-fold and the activated plants exhibited purple leaves similar to that of *PAP1* overexpressors. The *AVP1* gene expression was increased two- to five-fold in transgenic plants. In comparison to the wild type, *AVP1* activated plants had increased leaf numbers, larger single-leaf areas and improved tolerance to drought stress. The *AVP1* activated plants showed similar phenotypes to *AVP1* overexpressors. Therefore, the redesigned CRISPR/Cas9 activation system containing modified p65-HSF provides a simple approach for producing activated plants by upregulating endogenous transcriptional levels.

## Introduction

In recent years, type II clustered regularly interspaced short palindromic repeats (CRISPR) and CRISPR associated protein 9 (Cas9) have been developed into a robust RNA-guided gene editing system. The Cas9 is comprised of two endonuclease domains: HNH and RuvC-like domains. The HNH domain cleaves the DNA strand complementary to the guide RNA sequence, while the RuvC-like domain cuts the other non-complementary DNA strand through Watson-Crick base pairing by a sgRNA/Cas9 complex [1]. The complex of Cas9 and guide RNA results in a blunt-ended, double-stranded break upstream of the nearby NGG protospacer-adjacent motif (PAM) [2–4]. As a result of the double-stranded break, the type II CRISPR/Cas9 system effectively triggers non-homologous end joining (NHEJ) resulting in insertions or deletions (indels) which cause frameshift mutations in the coding region of a gene in eukaryotic systems [5–7].

CRISPR/Cas9 was originally employed to knockout target genes in various organisms. Recently, modifications to the Cas9 enzyme have extended the application of CRISPR/Cas9 to selectively activate target genes. Cas9 has undergone a series of function alterations by amino acid substitutions. After a single amino acid substitution of aspartic acid (D) to alanine (A) at the 10th amino acid in the RuvC-like domain, the Cas9 function is changed from endonuclease to nickase [1, 8]. Another amino acid substitution, a change from histidine (H) to alanine (A) at the 840th amino acid in the HNH domain of Cas9D10A, deactivates the nickase function. However, the point mutations that deactivate Cas9 do not disable the binding activities of Cas9 to the sgRNA and the target double strand DNA tertiary complex (S1 Fig) [1, 9]. When the point mutations have deactivated the Cas9 protein, the deactivated Cas9 (dCas9) (D10A/H840A) protein has the potential to become either an activator or a repressor. The dCas9 (D10A/H840A) turns into a functional transcription factor after a fusion with either an activator or a repressor domain, such as a fusion with the herpes simplex virus VP64 (tetramer of VP16) activation domain, the Krüppel-associated box (KRAB) repressor domain of KOX1, or the EAR-repressor domain (SRDX) from Arabidopsis SUPERMAN protein [10–12]. The fused dCas9VP64 was effective as a transcriptional activator with multiple sgRNAs rather than with a single sgRNA in Human Embryonic Kidney (HEK) 293T cells [8]

In order to reduce a cumbersome cloning machinery of multiple sgRNAs, a new CRISPR/Cas9 activation system was developed to maximize single sgRNA efficacy [13]. The new CRISPR/Cas9 was composed of dCas9VP64, a single gRNA, and the new additions, p65 transactivating subunit of NF-kappa B and human heat-shock factor 1 (HSF) activation domain. The p65 and HSF activation domains contributed to upregulated endogenous transcriptional levels independent from VP64 activation domain [13]. To bind the p65-HSF activating domains to single gRNA, a RNA-protein binding system was adopted, which was comprised of MS2 stem-loop and MS2 bacteriophage coat protein (MS2 protein). After being added into tetra-loop and stem-loop 2 in the middle of the sgRNA, the MS2 stem-loop recruits the MS2 protein, which binds to the MS2 stem-loop in sgRNA after fusion with p65-HSF domain (S1 Fig). The resulting CRISPR/Cas9 activation system with p65-HSF activators was tested with *Neurog2* gene in mouse Neuro-2a cells, which led to 12-fold upregulation of the *Neurog2* gene compared to the dCas9VP64 [13].

Here, we modified the CRISPR/Cas9 activation system with the p65-HSF activators to increase endogenous transcriptional levels in plants. This redesigned CRISPR/dCas9 activation system was tested with Arabidopsis *p**roduction of* *a**nthocyanin* *p**igment 1* (*PAP1*) and *A**rabidopsis thaliana* *v**acuolar H*^*+*^-*p**yrophosphatase* (*AVP1*). The *PAP1* encodes a MYB transcription factor. *PAP1* overexpression results in purple-colored Arabidopsis and tobacco plants due to the increased accumulation of anthocyanins [14]. The gene *AVP1* encodes a proton-pumping pyrophosphatase (H^+^-PPase) that pumps H^+^ across a mesophyll cell vacuole for acidification. The *AVP1* also controls auxin transport. Through proton pumping, *AVP1* increases the distribution and the abundance of the P-type H^+^-adenosine triphosphatase (P-ATPase) and the Pinformed 1 (PIN1) auxin efflux facilitator on the plasmamembrane [15]. *AVP1* overexpression results in an enlarged plant size, and improves nutrient uptake by increasing the abundance and activity of the plasmamembrane P-type H^+^-ATPase in a manner consistent with apoplastic pH alterations and rhizosphere acidification [16]. Both *PAP1* and *AVP1* overexpression phenotypes are clearly observable. Therefore, we used these two genes to test the CRISPR/Cas9 activation system in plants.

## Materials and methods

### Construction of binary vectors for *Avp*^*dcas9*^-*D* and *Pap*^*dcas9*^-*D*

**Fig 1:**
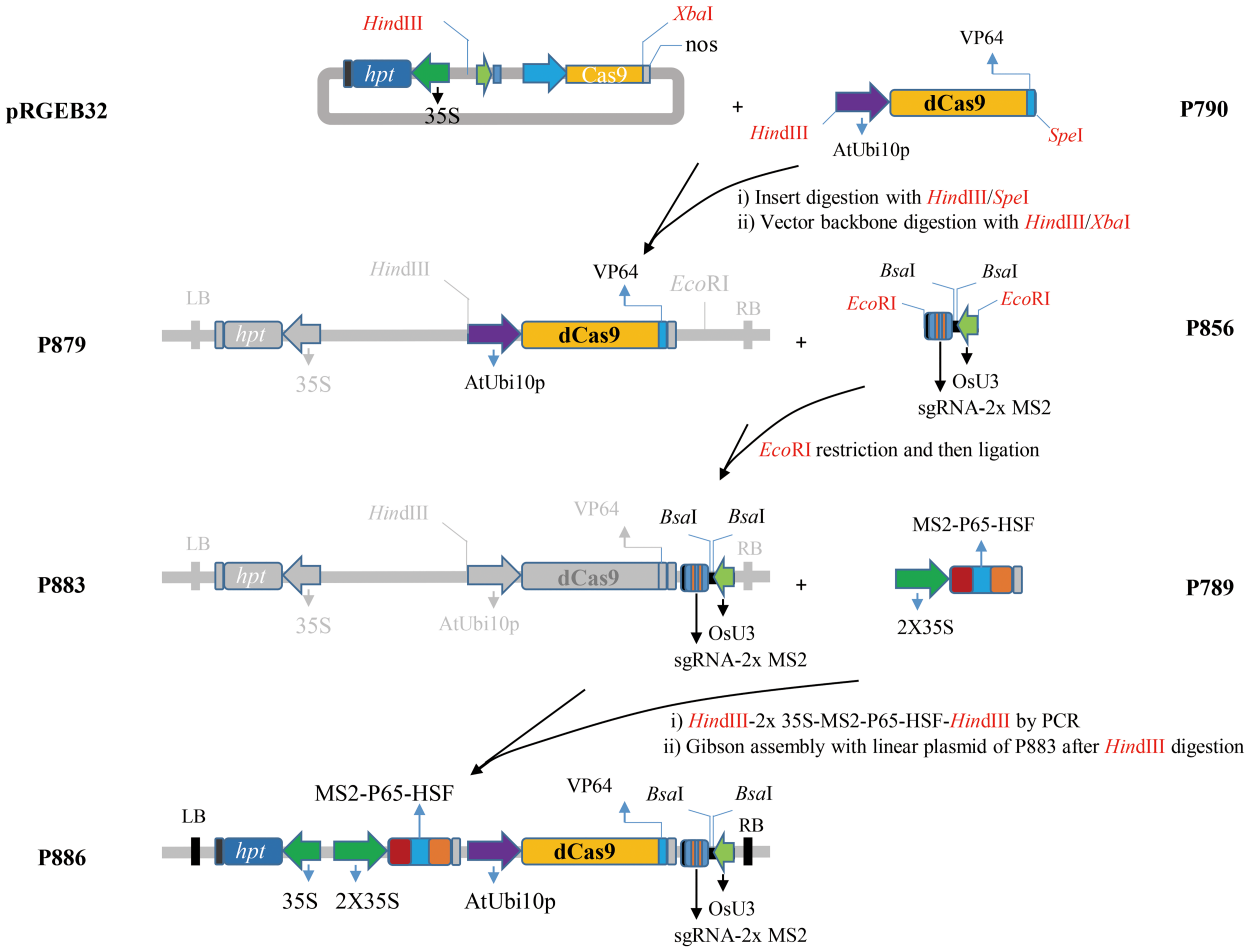
Schematic diagram of the procedure for constructing a binary vector (P886) for the transcriptional activation system.

Full-length *Streptococcus pyogenes* dCas9VP64 (D10A/H840A) was obtained by amplifying #61422 plasmid supplied through Addgene [13] with dCas9F1/dCas9R1 primer pairs (S1 Table), then cloned into pCR^®^-XL-TOPO^®^ sequencing vector (Invitrogen, Waltham, MA). The resulting construct was digested with *Kpn*I/*Spe*I restriction enzymes and cloned into sites of *Kpn*I and *Spe*I, located behind AtUbi10 promoter of pLC vector. The resulting AtUbi10-dCas9VP64 fragment (P790) was digested by *Hin*dIII and *Spe*I and ligated into a linear binary vector backbone from the pRGEB32, which was beforehand digested by *Hin*dIII and *Xba*I because the *Spe*I (A|CTAGT) and the *Xba*I (T|CTAGA) had the same 4 bp 5'-CTAG overhang. The resulting AtUbi10-dCas9VP64 vector (P879) had a single *Eco*RI enzyme site behind the *nos* terminator and was digested by *Eco*RI in order to ligate with *Eco*RI digested OsU3-sgRNA-2x MS2 stem-loop fragment (P856), which was obtained after replacement of the original human U6 promoter with the OsU3 promoter. An AtUbi10-dCas9VP64-nos:OsU3-sgRNA-2xMS2 (P883) was linearized by digestion with *Hin*dIII, and ligated through Gibson Assembly^®^ (NEB, Ipswich, MA) with 35S-MS2-p65-HSF-nos (P789), which was generated by PCR amplification with 32F35SF3/nosU10R3 primer pairs after replacement of the original human *EF*-*1α* promoter with the double 35S promoter (S1 Table). As a result, a working binary vector (P886) carrying 35S-Hyg^R^-nos, double 35S-MS2-p65-HSF-nos, AtUbi10-dCas9VP64-nos and OsUs3-sgRNA-2x MS2 was constructed (Fig 1). The P886 vector was digested by *Bsa*I (NEB, Ipswich, MA) and ligated with each target 20 bp dimers with GGCA/GGGT overhangs at flanking sites. In double targets, tRNA sequence was used to connect between two guide RNAs (S3 Fig) [17]. The target sgRNA sequences or cloning primers are listed on S1 Table.

### Plant materials and growth conditions

Arabidopsis ecotype Columbia was grown on MS media with 3% sucrose on 22°C with 70 μmol·m^-2^·s^-1^ light intensity in a 16/8 h light/dark photoperiod chamber, and were then transtered to soil at 22°C with 85 μmol·m^-2^·s^-1^ light intensity in a 16/8 h light/dark photoperiod chamber in order to obtain seeds. *Pap1*^*dcas9*^*-D* plants on soil were grown under 250 μmol·m^-2^·s^-1^ light intensity that were used for the identification of the *PAP1* activation tagging mutant [14].

### Protoplast transfection assay

Fresh leaves of Arabidopsis plants grown under a short day condtion (10/14 h) were sliced with a razor blade. Polyethylene glycol (PEG)-mediated transformation was performed as described previously [18] with minor modifications. The sliced leaves were incubated in a 20 ml enzyme solution (1.5% [w/v] Cellulose RS, 0.3% [w/v] Macerozyme, 0.4 M mannitol, 20 mM MES, pH 5.7, 1 mM CaCl_2_, and 0.1% [w/v] bovine serum albumin) for 4 h in the dark with gentle shaking. After adding an equal volume of W5 solution (154 mM NaCl, 125 mM CaC1_2_, 5 mM KCl, and 2 mM MES, pH 5.7) and harvesting the cells, the protoplasts were resuspended in 2 ml Mmg solution (0.4 M mannitol, 15 mM MgCl_2_, and 4 mM MES, pH 5.7). For transformation, 10 μg of each vector plus 200 μl of 40% PEG solution (0.4 M mannitol, 100 mM CaCl_2_, 40% [w/v] PEG 4000) was added to a 1.7 ml tube containing 200 μl Mmg with 2 x 10^7^ protoplasts for 10 min. The protoplasts were washed with two volumes of W5 solution and then resuspended in an incubation solution (0.4 M mannitol, 4 mM MES, pH 5.7, and 4 mM KCl). They were then incubated for 48 hours at 25°C in the dark.

### qRT-PCR measurement of endogenous expression

The leaves of 1-month-old T1 Arabidopsis plants were used for a total RNA extraction. Total RNA was extracted by using 1ml TRI Reagent^®^ (Sigma, MO, USA) and a 0.1 ml of 1-bromo-3-chloropropane (MRC, OH, USA) according to the manufacturer’s instructions. Sample mixtures were placed at room temperature for 5 minutes after shaking well. The sample mixtures were centrifuged for 15 min at 4°C. After transfering 400 μl supernants, 300 μl isopropanol was added into new 1.7 ml tubes. The 1.7 ml tubes containing supernants were centrifuged for 8 min at 4°C. The resulting pellets were washed with 75% EtOH. Two micrograms of RNA were reverse transcribed using reverse transcriptase of the Superscript III Kit (Invitrogen) after treatment with TURBO^™^ DNase I (Ambion, Austin, TX). The Ct values of qRT-PCR were generated by ABI PRISM 7900 HT sequence detection system (Applied Biosystems). *Actin 2* was used as an endogenous control for normalization of qRT-PCR. Changes in gene expression were calculated via the 2^−ΔCT^ method. The qRT-PCR experiments had two biological replicates and three technical replicates.

## Results

### Structure-guided design of CRISPR/dCas9 complex for plants

The transcriptional activation CRISPR/dCas9 system was designed by the fusion of the VP64 (tetramer of VP16) transactivation domain to the C-terminus of the dCas9 protein [6, 19]. In order to achieve transcriptional activation in plants, we constructed a working binary vector. The first step in creating a working binary vector was the preparation of a binary vector backbone and the three inserts: dCas9VP64, sgRNA with MS2 stem-loop and MS2-p65-HSF activators (see Materials and methods). Second, we introduced the AtUbi10 promoter in front of dCas9, and AtUbi10-dCas9 (P790) was created. Third, the digested P790 was inserted into the pRGEB32 binary vector backbone, which was previously digested with *Hin*dIII and *Xba*I, and the AtUbi10-dCas9 binary vector (P879) was constructed. Fourth, the P879 was digested by *Eco*RI and ligated with *Eco*RI-digested OsU3-sgRNA-2xMS2 (P856) to generate a new AtUbi10-dCas9VP64:OsU3-sgRNA-2xMS2 binary vector (P883). Then, 2x35S-MS2-p65-HSF (P789) was inserted into P883 through Gibson assembly. As a result, a working binary vector (P886)–carrying 35S-Hyg^R^, 2x35S-MS2-p65-HSF, AtUbi10-dCas9VP64, and OsUs3-sgRNA-2x MS2 –was produced (Fig 1).

### Construction for *PAP1* and *AVP1* activation vectors

The first target gene, *PAP1*, has a 747 bp coding sequence with 3 exons and 48 bp 5' untranslated region. The core promoter region of 400 bp to 48 bp upstream of ATG site was chosen as the preferential target for activating transcription. The sgRNAs were designed upstream of protospacer-adjacent motif (PAM); they confer sequence specificity and close to the transcriptional start site (TSS) in the core promoter region. The designed sgRNAs have 20 bp targets from -69th to -50th (P902) and from -102nd to -83rd (P903) upstream of the ATG site, respectively (S2 Fig and S2 Table).

The second target gene, *AVP1*, has a 2,313 bp coding seqeunce with 8 exons and 125 bp untranslated region. The preferential target was from 400 bp to 126 bp upstream of ATG site of *AVP1*. sgRNAs were designed using 20 bp from -191st to -172nd (P900) and 20 bp from -235th to -216th (P901) from ATG site, respectively (S2 Fig and S2 Table).

Furthermore, sgRNAs with double target sites were also designed. Two 20 bp sequences at -172nd and at -216th upstream of the *AVP1* gene were used for *AVP1* activation (P904). Two 20 bp targets at -50th and at -83rd upstream of *PAP1* were used for *PAP1* activation (P905) (S3 Fig and S2 Table).

### Evaluation for activation of endogenous gene transcription through transient assay

**Fig 2:**
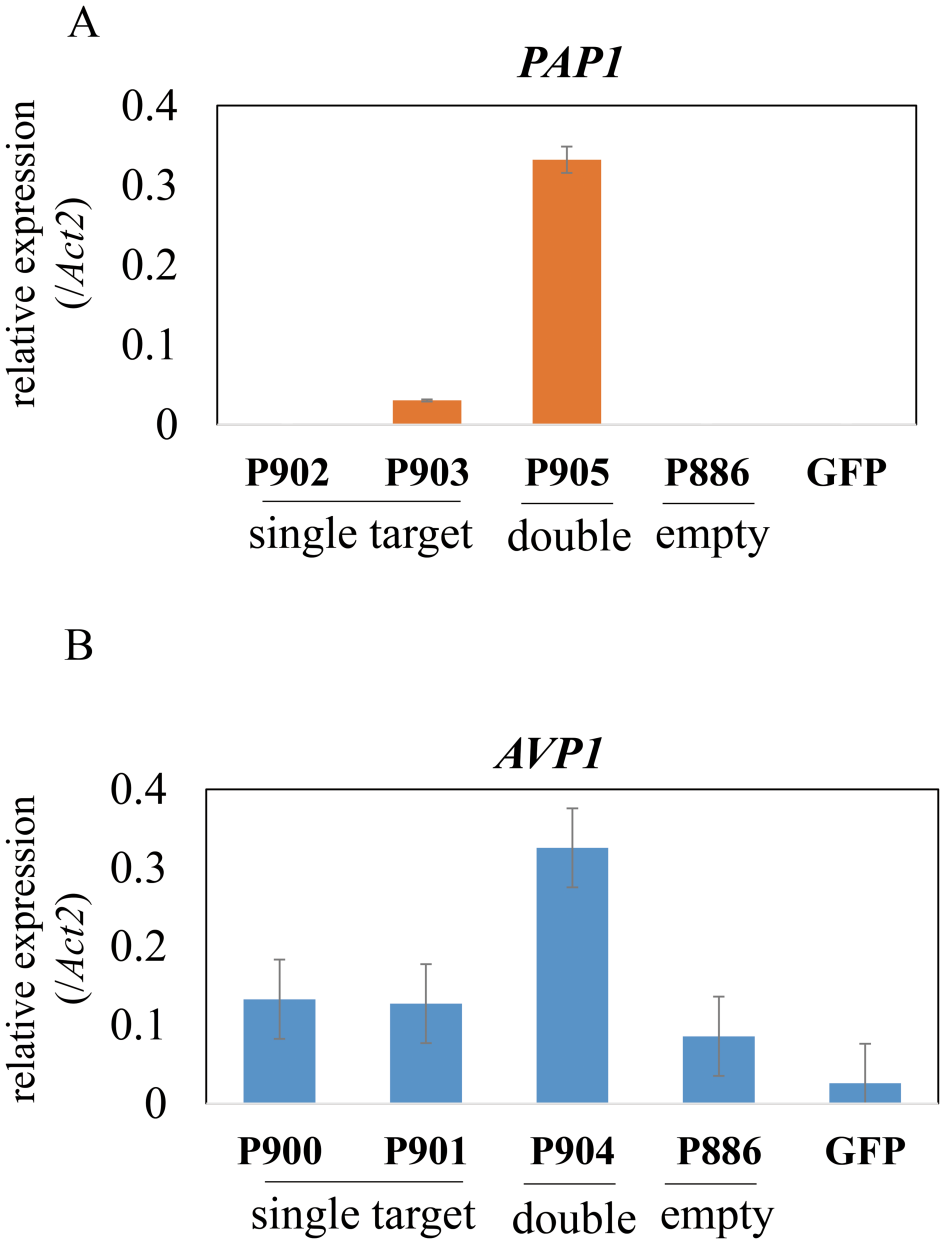
qRT-PCR analysis of transient expression levels of *PAP1* (A) and *AVP1* (B) constructs in Arabidopsis mesophyll protoplasts. *PAP1* (P902 and P903 each carrying a single target; P905 carrying double targets), *AVP1* (P900 and P901 each carrying a single target; P904 carrying double targets), empty vector (P886) and GFP (35S-GFP). *Actin 2* was used as an endogenous control (value = 1).

To quickly test whether the activation system was able to increase endogenous gene expression, we carried out a transient assay using Arabidopsis mesophyll protoplasts. The constructs, *AVP1* (P900, P901 and P904), *PAP1* (P902, P903 and P905), an empty vector (P886) control, and GFP control, were transfected into Arabidopsis mesophyll protoplasts. Primers for analyzing the expression levels were designed to span an exon to exon junction behind the target sites so that the target region did not affect PCR performance (S2 Fig and S1 Table). The *PAP1* expression was detected only in protoplast transfected with constructs P903 and P905, while no signal was detected in other vectors (Fig 2A). The *AVP1* construct P904 led to a two-fold higher expression level than the empty (P886) and the GFP vectors while *AVP1* constructs P900, P901 showed no significant difference compared with the empty and the GFP vectors (*p* > 0.05) in qPCR analysis (Fig 2B).

### Activation of endogenous genes through stable transformation

**Fig 3:**
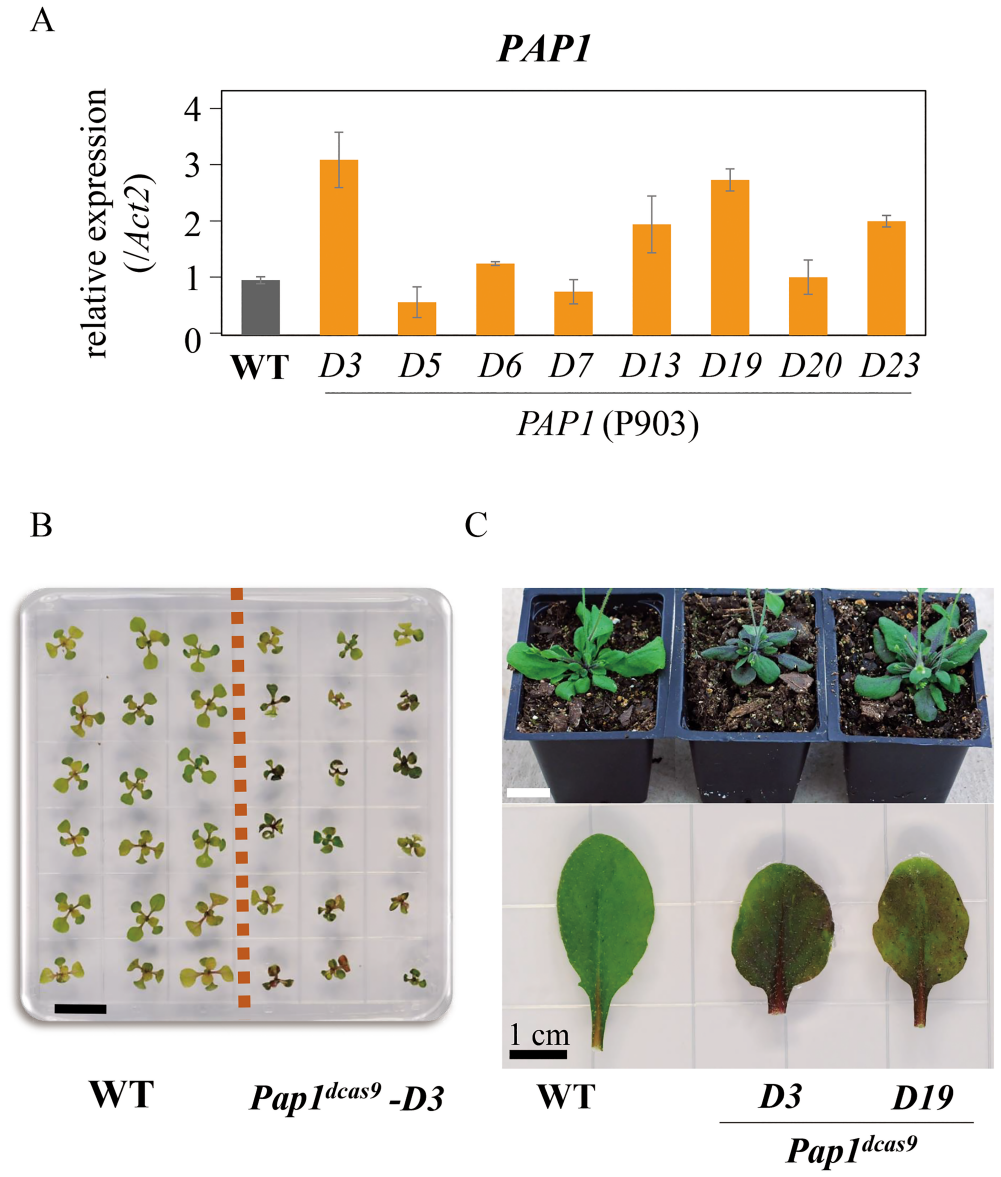
Expression level and phenotype of *PAP1* activation plants. (A) qRT-PCR analysis of expression levels of *PAP1* (P903) T1 transgenic plants under 250 μmol·m^-2^·s^-1^ light intensity. (B) Seedling phenotypes of *Pap1*^*dcas9*^ -*D* on culture media. (C) One-month-old plants showing purple color in growth chamber (top), adaxial side showing purple color (bottom). *Actin 2* was used as an endogenous control. Scale bar, 1cm.

Based on the results of above transient assay, selected *PAP1* (P903, and P905) and *AVP1* (P904) constructs were used for stable transformation. Transgenic Arabidopsis plants were produced by *Agrobacterium*-mediated transformation. T1 plants with increased *PAP1* expression levels were identified (*D3*, *D13*, *D19*, and *D23* of P903 and *D31*, *D32*, *D33*, *D34*, and *D35* of P905; Fig 3A and S4 Fig). The activated *PAP1* T2 plants carrying the single target construct P903 showed a purple color in the seedlings (Fig 3B), while the activated *PAP1* T2 plants carrying the double target construct P905 showed not only a purple color but also dwarf seedling (S4 Fig). We named the *PAP1*-activated plants *P**roduction of* *a**nthocyanin* *p**igment 1 by dCas9* *D**ominant*, abbreviated as *Pap1*^*dCas9*^ *-D*. The purple color was observed consistantly in seedlings and after being transferred to soil under 250 μmol·m^-2^·s^-1^ light condition (Fig 3C).

**Fig 4:**
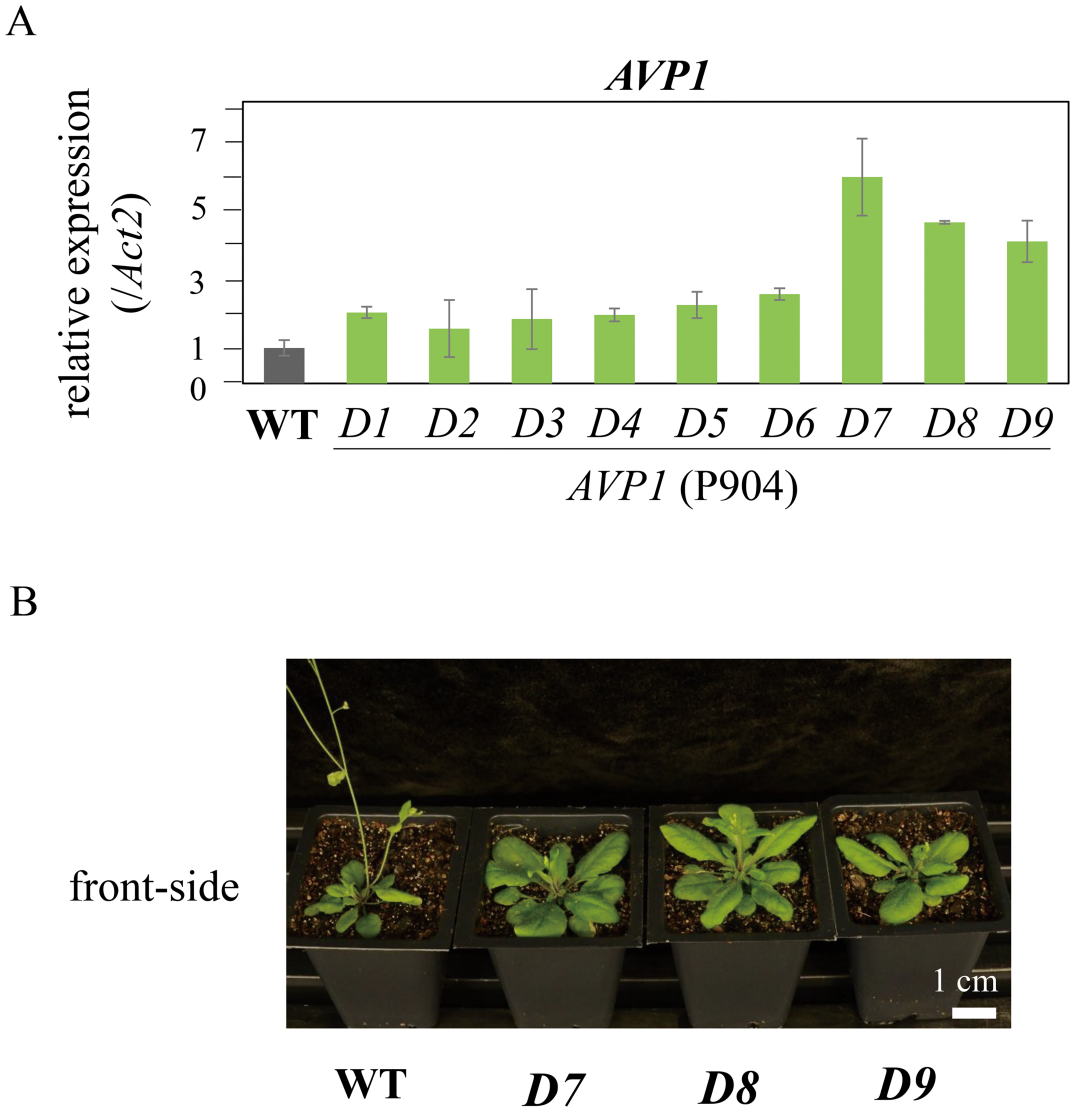
Expression level and phenotype of *AVP1* activation plants. (A) qRT-PCR analysis of expression levels of *AVP1* (P904) transgenic plants. (B) Phenotype of *AVP1* (P904) plants. *Actin 2* was used as an endogenous control.

**Fig 5:**
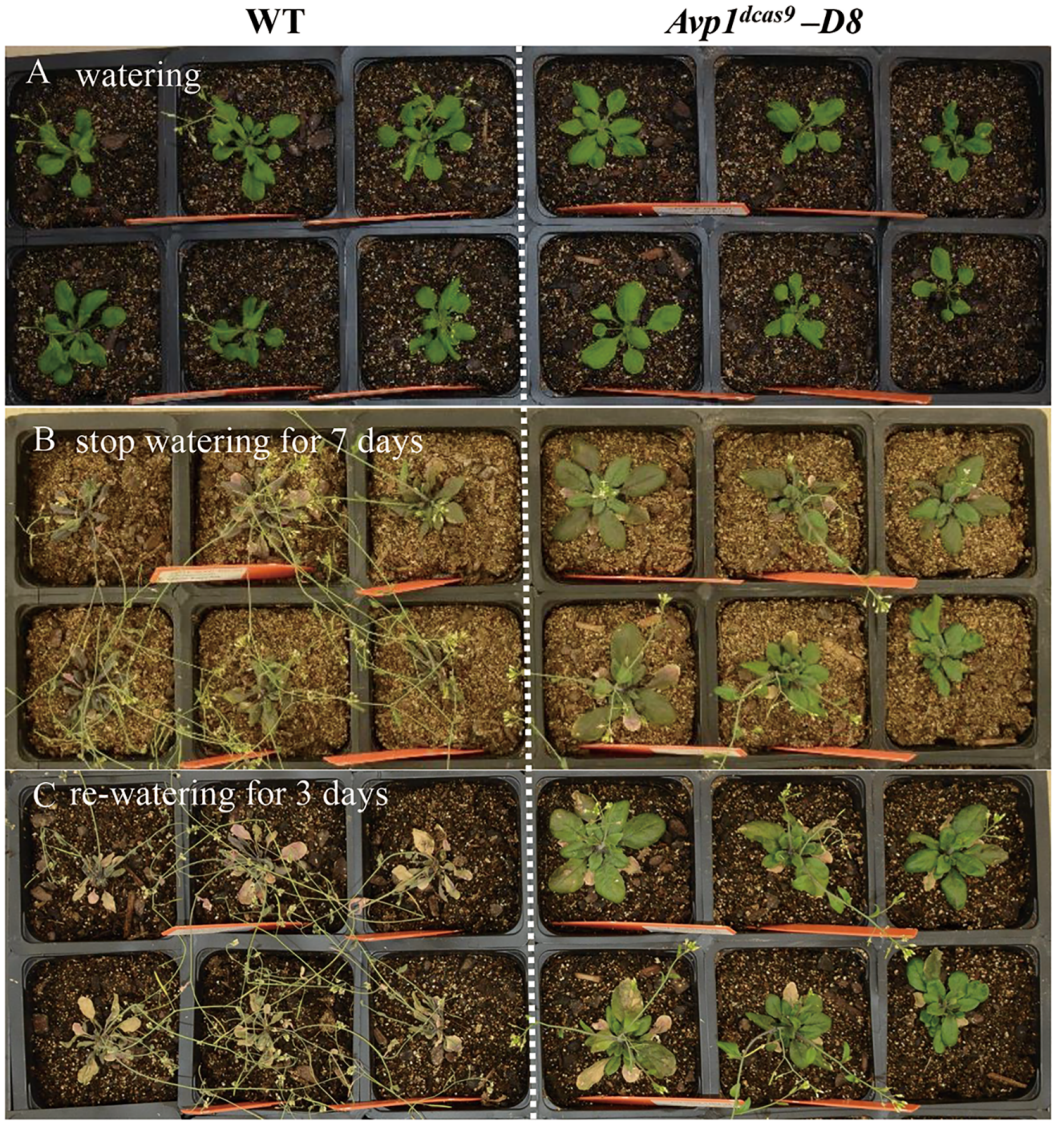
Phenotype of WT and *AVP* activation (*Avp1*^*dcas9*^ -*D*) T2 plants before or after drought stress. (A) Plants grown under regular conditions. (B) Watering was stopped for seven days. (C) Three days after re-watering the plants.

The *AVP1* (P904) carrying on two targets of *AVP1* provided two- to five-fold increase in *AVP1* transcriptional levels. The T1 plants with relatively high expression levels (*D7*, *D8*, and *D9*) showed larger plant size than the wild type (Fig 4A and 4B). The flowering time of these plants was delayed by six days, the number of rosette leaves increased up to four leaves, and the single-leaf area was increased by1.6 to two-fold in the eighth leaf (Table 1). We named the *AVP1*-activated plants *A**rabidopsis thaliana* *v**acuolar H+*-*p**yrophosphatase 1 by dCas9* *D**ominant* (*AVP1*^*dCas9*^ *-D*). The line *D8* T2 plant was tested for drought stress tolerance by discontinuing watering for seven days. The wild type wilted and died after seven days without watering while the activated line (*D8*) remained green. The activated line (*D8*) restored growth after re-watering, while the wild type failed completely to recover (Fig 5A–5C).

**Table 1:** Flowering time, rosette leaf number and single-leaf area of *AVP1* (P904) activation plants.

	WT	P904-*D7*	P904-*D8*	P904-*D9*
Flowering time (days)	18 ± 0.9	25 ±1.5	24 ± 1.7	25 ± 1.9
Rosette leaf No.	10 ± 0.6	14 ± 1.7	14 ± 1.9	14 ± 1.3
Single-leaf area (cm^2^)	0.9 ± 0.1	2.1 ± 0.3	2.2 ± 0.2	1.6 ± 0.1

WT and *Avp*^*dcas9*^ (P904) *-D7*, *-D8* and *-D9* (n = 15/line of T2 plants) were grown in soil. The area of single-leaf at the eighth leaf (n = 6). Mean ± SD.

## Discussion

### Mimicry of *PAP1* and *AVP1* overexpressions

Overexpression of the *PAP1* showed a purple color under high light conditions. High light was required for induction of *PAP1* and basic Helix-Loop-Helix (bHLH) transcription factors in anthocyanin biosynthesis pathway [20, 21]. Thereby, *PAP1* activated plants were screened for the purple phenotype under a high light (250 μmol·m^-2^·s^-1^). *PAP1* gene expression was measured under high light. A three-fold higher expression of *PAP1* led to a bright purple color in *Pap1*^*dCas9*^*-D* transgenic plants similar to *PAP1* overexpressors. In addtion, the activated plants (P905) carrying double target sgRNAs had various phenotypes, including growth retardation, lethality and a purple color at seedling stage. According to *PAP1* activation experiments, in order to produce suitable activated plants, different target sites should be tested to avoid off target phenotypes.

The phenotypes of *AVP1* overexpressors presented 3 to16 more leaves and 40% to 60% larger single-leaf area than the wild type (Li et al. 2005). The *AVP1* overexpressors also showed tolerance to salt and drought stress because they accumulated more Na^+^ and K^+^ in the overexpressing leaves than the wild type [16]. The *Avp1*^*dcas9*^ -*D* plants showed two- to five-fold increases in gene expression, which resulted in additional four leaves, a two-fold increased single-leaf area and enhanced drought tolerance. The resulting phenotypes of the *Avp1*^*dcas9*^ -*D* plants were similar to *AVP1*-*1* overexpressors. Therefore, the modified CRISPR/Cas9 activation system with p65-HSF activators provides a simple approach for producing activated plants through upregulating endogenous transcriptional levels.

### The performance of redesigned activation system

The previous CRISPR/dCas9 has proven to increase transcriptional expressions in plant cells and plants [11, 19]. Unfortunately, the reported increased gene expression by the CRISPR/dCas9 failed to result in overexpression phenotypes in Arabidopsis [19]. *PAP1*, utilized in the current study, was previously used for activation by a CRISPR/dCas9 system by Lowder et al. (2015). Three sgRNAs from core promoter of *PAP1* were used to increase transcription levels; the *PAP1* gene expression was significantly increased, but the purple color was not observed in the *PAP1* activation lines (Lowder et al. 2015). In contrast, our *Pap1*^*dcas9*^-*D* plants exhibited purple color similar to *PAP1* overexpression [14]. Our *Avp1*^*dcas9*^-*D* had larger leaf areas and increased leaf numbers similar to *AVP1* overexpression [15]. Therefore, our study demonstrated, for the first time, that CRISPR/dCas9 system can be effectively used for gene activation in plants. The major difference between the current study and the previouly reported CRISPR/dCas9 activation system is the use of additional p65-HSF activators with sgRNA.

### A potential activation system to generate novel gain-of-function mutations

In the application of CRISPR/dCas9 for plant development, endogenous transcription levels of the target gene reproduce the spatial and the temporal gene expression profiles of the wild type. In contrast, ectopic expression, using constitutive promoters and full-length cDNAs, alters endogenous transcription expression pattern. The use of ectopic expression overlooks various splicing patterns in different tissues and developmental stages. As a result, ectopic transcripts mask alternative transcripts, which may lead to isoenzyme activity, wrong-interaction partners, mis-localization, and instablility of the original protein [22, 23]. Therefore, activation through the CRISPR/dCas9 system compensates for the shortcomings of commonly used ectopic overexpression. Furthermore, it is relatively simple to construct activation vectors because the CRISPR/dCas9 system requires only a 20 bp sequence in the core-promoter, regardless of cDNA full-length size and availabilty.

## Supporting information

S1 Fig Schematic diagram of two different CRISPR/dCas9 transcriptional activation systems. (A) dCas9-VP64 and guide RNA. (B) dCas9-VP64 and guide RNA 2x MS2, MS2-p65-HSF.
(TIF)

S2 Fig Schematic diagrams of *PAP1* and *AVP1* gene structures. (TIF)

S3 Fig Sequence information for the construction of candidate targets. The P886 vector was digested by *Bsa*I and ligated with each target 20 bp dimers with GGCA/GGGT overhangs at flanking sites. In double targets, tRNA sequence was used to connect between two guide RNAs. Dark bule letters: OsU3; green letters: sgRNA scaffold; bold and blue letters: target sites; grey letters: tRNA; red letters,:4 bp over hangs by B*sa*I.
(TIF)

S4 Fig Expression level and phenotype of *PAP1* (double target) activation plants. (A) qRT-PCR analysis of *PAP1* (P905) seedlings under 85 μmol·m^-2^·s^-1^ light intensity. (B) Phenotype of *PAP1* (P905) seedlings -*D31*, -*D32*, -*D33*, -*D34* and -*D35* on media under 150 μmol·m^-2^·s^-1^ light intensity. (C) Phenotype of *PAP1* (905) -*D32* seedlings under 150 μmol·m^-2^·s^-1^ light intensity. (D) Phenotype of *PAP1* (905) -*D33* seedlings under 150 μmol·m^-2^·s^-1^ light intensity. Red arrows in (C) and (D) indicate small or lethal seedlings. *Actin 2* was used as an endogenous control.
(TIF)

S1 Table Primer sequences used in this study. (XLSX)

S2 Table Construct information for target genes, PAM sequences and direction. (XLSX)
