# Supplementary figures and images for: RNA-guided transcriptional activation via CRISPR/dCas9 mimics overexpression phenotypes in Arabidopsis
Source: PLoS One. 2017 Jun 16;12(6):e0179410. doi: 10.1371/journal.pone.0179410 (PMC5473554; doi:10.1371/journal.pone.0179410)

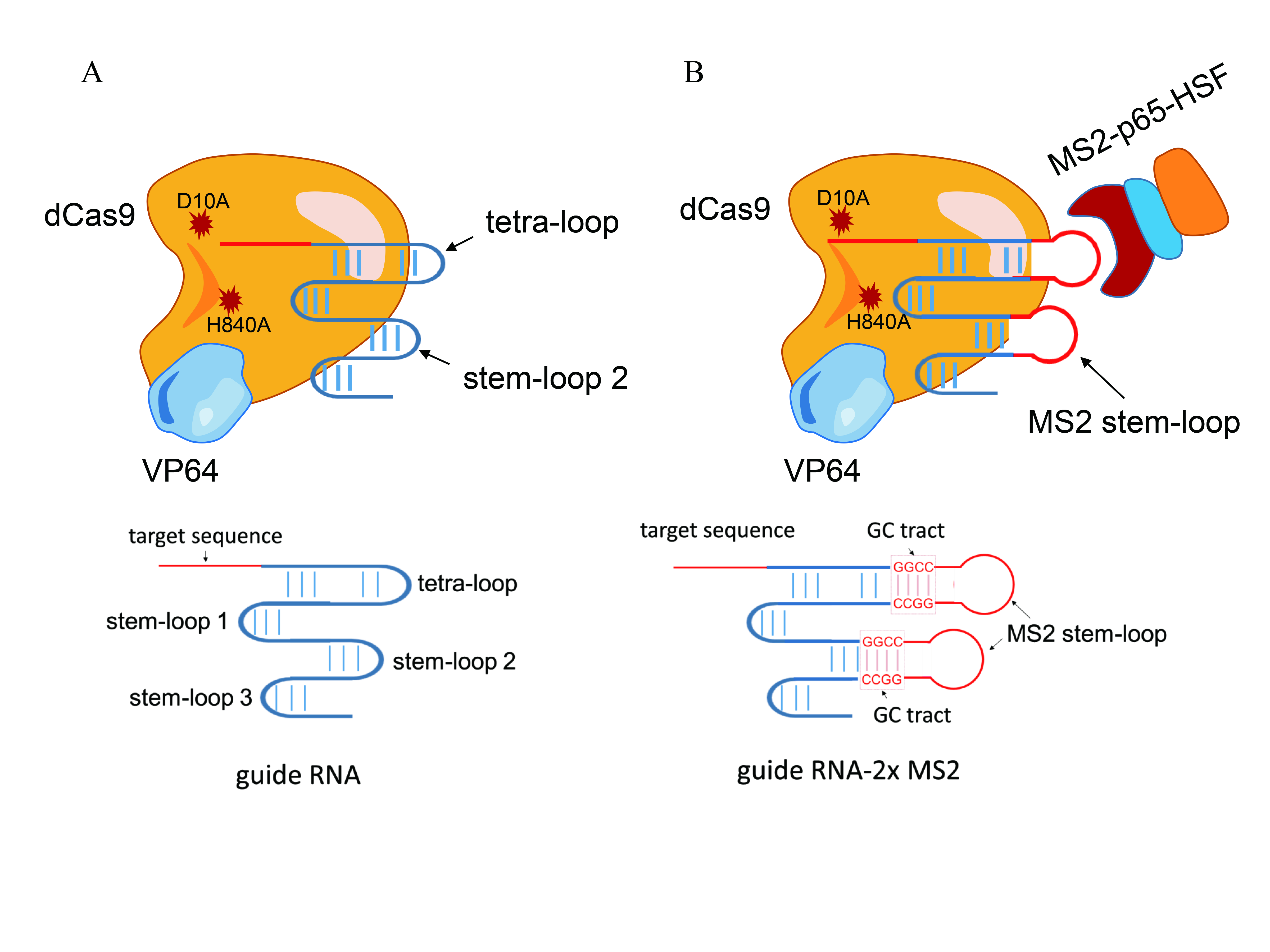

Supplement: S1 Fig — (A) dCas9-VP64 and guide RNA. (B) dCas9-VP64 and guide RNA 2x MS2, MS2-p65-HSF. (TIF) [file pone.0179410.s001.tif]

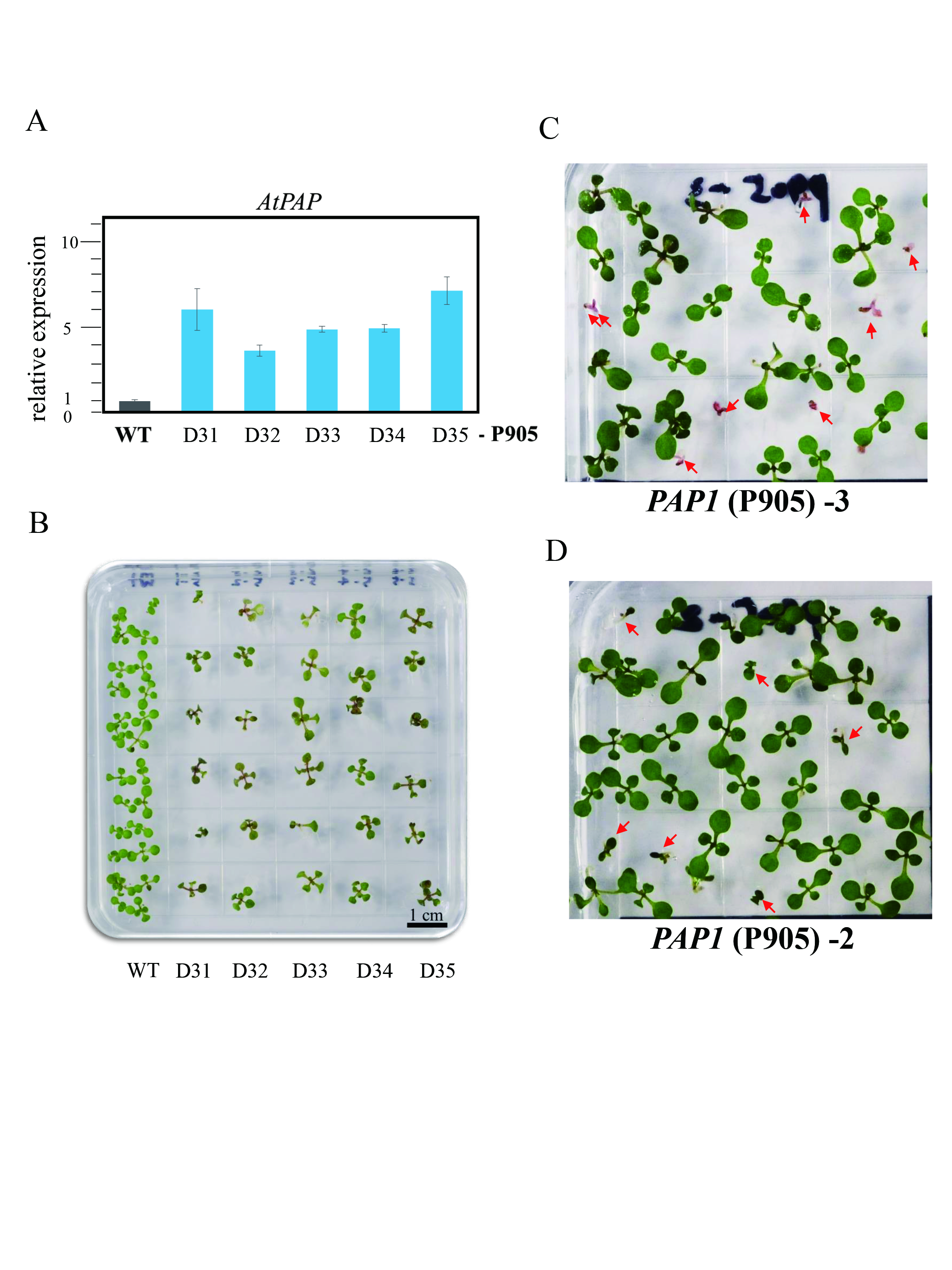

Supplement: S4 Fig — (A) qRT-PCR analysis of PAP1 (P905) seedlings under 85 μmol·m-2·s-1 light intensity. (B) Phenotype of PAP1 (P905) seedlings -D31, -D32, -D33, -D34 and -D35 on media under 150 μmol·m-2·s-1 light intensity. (C) Phenotype of PAP1 (905) -D32 seedlings under 150 μmol·m-2·s-1 light intensity. (D) Phenotype of PAP1 (905) -D33 seedlings under 150 μmol·m-2·s-1 light intensity. Red arrows in (C) and (D) indicate small or lethal seedlings. Actin 2 was used as an endogenous control. (TIF) [file pone.0179410.s004.tif]
